# Single-cell analysis unveils activation of mast cells in colorectal cancer microenvironment

**DOI:** 10.1186/s13578-023-01144-x

**Published:** 2023-11-29

**Authors:** Zhenyu Xie, Liaoran Niu, Gaozan Zheng, Kunli Du, Songchen Dai, Ruikai Li, Hanjun Dan, Lili Duan, Hongze Wu, Guangming Ren, Xinyu Dou, Fan Feng, Jian Zhang, Jianyong Zheng

**Affiliations:** 1 The State Key Laboratory of Cancer Biology, Department of Gastrointestinal Surgery, Xijing Hospital, Fourth Military Medical University, 169 Changle Road, Xi’an, Shaanxi 710032 China; 2 Department of Surgical Oncology and General Surgery, The First Hospital of China Medical University, Shenyang, 110016 China; 3 Key Laboratory of Precision Diagnosis and Treatment of Gastrointestinal Tumors, Ministry of Education, Shenyang, 110016 China; 4 Xi’an Medical University, Xi’an, Shaanxi 710021 China; 5 The State Key Laboratory of Cancer Biology, Department of Biochemistry and Molecular Biology, The Fourth Military Medical University, 169 Changle Road, Xi’an, Shaanxi 710032 China

**Keywords:** Mast cells, Colorectal cancer, Heterogeneity, Activation, Single-cell analysis, Prognosis

## Abstract

The role of mast cells (MCs) in colorectal cancer (CRC) remains unclear, and a comprehensive single-cell study on CRC MCs has not been conducted. This study used a multi-omics approach, integrating single-cell sequencing, spatial transcriptomics, and bulk tissue sequencing data to investigate the heterogeneity and impact of MCs in CRC. Five MC signature genes (*TPSAB1*, *TPSB2*, *CPA3*, *HPGDS*, and *MS4A2*) were identified, and their average expression was used as a marker of MCs. The MC density was found to be lower in CRC compared to normal tissue, but MCs in CRC demonstrated distinct activation features. Activated MCs were defined by high expression of receptors and MC mediators, while resting MCs had low expression. Most genes, including the five MC signature genes, were expressed at higher levels in activated MCs. The MC signature was linked to a better prognosis in both CRC and pan-cancer patient cohorts. Elevated *KITLG* expression was observed in fibroblasts and endothelial cells in CRC samples compared to normal tissue, and co-localization of MCs with these cell types was revealed by spatial transcriptome analysis. In conclusion, this study finds decreased MC density in CRC compared to normal tissue, but highlights a shift in MC phenotype from *CMA1*^high^ resting cells to activated *TPSAB1*^high^, *CPA3*^high^, and *KIT*^high^ cells. The elevated *KITLG* expression in the tumor microenvironment’s fibroblasts and endothelial cells may activate MCs through the *KITLG*-*KIT* axis, potentially suppressing tumor progression.

**Supplementary Information:**

The online version contains supplementary material available at 10.1186/s13578-023-01144-x.

## Introduction

Colorectal cancer (CRC) is a prevalent and lethal type of cancer, ranking as the third most common malignancy and the second leading cause of cancer-related death worldwide [1]. Despite the recent advancements in immunotherapy, its efficacy in treating CRC remains limited, with only a small subset of patients with mismatch repair deficiency (dMMR) or high levels of microsatellite instability (MSI-H) experiencing positive outcomes from immune checkpoint blockade (ICB) [2–5]. To address this challenge, it is crucial to gain a deeper understanding of the complex interactions between different cells and molecules in the CRC tumor microenvironment (TME) and identify new targets for immunotherapy.

Mast cells (MCs) are a type of immune cell that play a crucial role in the body’s response to allergens and in defending against pathogens [6, 7]. Upon activation, MCs release a variety of mediators, including proteases, cytokines, histamine, and lipid mediators, which have been implicated in the development of various diseases, including allergies, asthma, autoimmune disorders, and infections [6, 8, 9]. However, despite extensive research, the role of MCs in cancer, including CRC, remains controversial [9–11]. While some studies suggest a pro-tumorigenic role for MCs in CRC [12–15], others report an inhibitory effect [16–18]. Further investigation is necessary to gain a comprehensive understanding of the role of MCs in CRC.

The growth of single-cell sequencing techniques has revolutionized biological research by providing a detailed understanding of molecular and functional heterogeneity within individual cells [19, 20]. In the context of CRC, single-cell sequencing has been widely applied to study the molecular and functional profiles of immune cells in TME, such as T cells and myeloid cells [21, 22]. However, despite the growing body of research in this area, the molecular and functional heterogeneity of MCs within the TME of CRC remains unexplored.

This study aims to fill this gap by leveraging single-cell sequencing technologies to investigate the heterogeneity of MCs in the TME of CRC. Our findings represent the first identification of MC activation in CRC, revealing potential mechanisms behind this activation and the protective role of MCs in prognosis. This study provides valuable insights into the complex interactions between cancer and the immune system, and has implications for the development of novel therapeutic strategies for CRC.

## Methods

### Material

The study utilized various public datasets, including one Spatial Transcriptomics (ST) dataset, three single-cell RNA-sequencing (scRNA-seq) datasets, and 11 bulk RNA-sequencing (bulk RNA-seq) datasets (Table 1). The bulk RNA-seq datasets comprise high-throughput sequencing data from the TCGA-CRC and ten Gene Expression Omnibus (GEO) microarray datasets (GSE20842, GSE20916, GSE39582, GSE41258, GSE44076, GSE44861, GSE68468, GSE83889, GSE87211, and GSE106582). The transcriptome data and clinical information from the TCGA-CRC were obtained from UCSC Xena (http://xena.ucsc.edu/), while those from the TCGA pan-cancer cohort were obtained from the National Cancer Institute Cancer Research Data Commons (https://gdc.cancer.gov/about-data/publications/pancanatlas) and UCSC Xena. Transcriptome and clinical information from the GEO datasets were acquired from the GEO database (https://www.ncbi.nlm.nih.gov/geo/).

**Table 1:** Sources of the ST, scRNA-seq, and bulk RNA-seq datasets

Deposited Data	Platform	Identifier
Human CRC ST dataset	10x Genomics	http://www.cancerdiversity.asia/scCRLM
Human CRC scRNA-seq dataset	10x Genomics	https://www.synapse.org/#!Synapse:syn26844071/
Human CRC scRNA-seq dataset	10x Genomics	GEO: GSE178341
Human CRC scRNA-seq dataset	10x Genomics	GEO: GSE164522
Human CRC bulk RNA-seq dataset	Illumina Hiseq	TCGA
Human CRC bulk RNA-seq dataset	GPL4133	GEO: GSE20842
Human CRC bulk RNA-seq dataset	GPL570	GEO: GSE20916
Human CRC bulk RNA-seq dataset	GPL570	GEO: GSE39582
Human CRC bulk RNA-seq dataset	GPL96	GEO: GSE41258
Human CRC bulk RNA-seq dataset	GPL13667	GEO: GSE44076
Human CRC bulk RNA-seq dataset	GPL3921	GEO: GSE44861
Human CRC bulk RNA-seq dataset	GPL96	GEO: GSE68468
Human CRC bulk RNA-seq dataset	GPL10558	GEO: GSE83889
Human CRC bulk RNA-seq dataset	GPL13497	GEO: GSE87211
Human CRC bulk RNA-seq dataset	GPL10558	GEO: GSE106582

### Cell culture

The mouse CRC cell line MC38 was maintained in our lab (Xian, China). The mouse mast cell line P815 was obtained from pricella (Wuhan, China). Both cells were cultured in DMEM medium (Gibco, Thermo Fisher Scientific, Cambridge, MA, USA), containing 10% fetal bovine serum (Oricell; Guangzhou, China), 100 µg/ml streptomycin, and 100 U/ml penicillin in the medium (HyClone; Logan, Utah, USA). Both cells were incubated in a humidified incubator at 37℃ with 5% CO_2_.

### Total RNA extraction and qRT-PCR

Trizol reagent (Invitrogen, Waltham, MA, United States) was used to isolate and extract total RNA from P815 cell line. The obtained RNA was then reverse transcribed into cDNA using the PrimeScript RT Reagent Kit (TaKaRa, Tokyo, Japan). qRT-PCR was then employed using the SYBR Premix Ex Taq II Kit (TaKaRa, Tokyo, Japan) to measure the expression levels of KIT. GAPDH was set as the internal standard. The relative mRNA expression was calculated using the 2^−ΔΔCt^ method. The primer sequences are provided as following. *KIT* forward: 5’-GGCCTCACGAGTTCTATTTACG-3’; reverse: 5’-GGGGAGAGATTTCCCATCACAC-3’; *GAPDH* forward: 5′-GGTGAAGGTCGGTGTGAACG-3′; reverse: 5′-CTCGCTCCTGGAAGATGGTG-3′.

### Western blot assay

For Western blot assays, we analyzed both human CRC tissue samples and mouse cell (P815) lysates. Human samples were obtained from Xijing Hospital, and proteins were isolated using RIPA lysis buffer supplemented with a protease inhibitor cocktail. The primary antibodies used for human samples were mouse anti-human *TPSAB1* (Thermo Scientific, Cat#ab2378, 1:50) and β-actin (#3700S, Cell Signaling Technology, 1:1000). In contrast, mouse cell lysates were prepared and their protein concentrations determined using a BCA kit. The primary antibodies for mouse cells were anti-KIT (#PA6364, abmart, Shanghai, China, 1:500) and anti-β-actin (#3700S, Cell Signaling Technology, 1:1000). Both human and mouse samples underwent 10% SDS-PAGE and were transferred onto either nitrocellulose or nylon membranes. After incubation at 4 °C overnight with primary antibodies, membranes were treated for one hour at 37 °C with HRP-conjugated secondary antibodies specific to either mouse IgG or rabbit IgG. Visualization and quantification of protein bands were performed using enhanced chemiluminescence and ImageJ software, respectively.

### CCK-8 assay

P815 cells were pre-incubated in DMEM supplemented with different concentrations of KITLG (0ng/ml, 50ng/ml, or 200ng/ml). The medium supernatant was collected for subsequent culture of MC38 cells. In a 96-well plate, 2 × 10^3^ MC38 cells were seeded in each well with 100 µl of medium. At 0, 24, 48, 72, and 96 h, following the removal of the original medium, a mixture of CCK-8 solution (TransDetect Cell Counting Kit, Transgene, Beijing, China) and fresh medium (without FBS) in a 1:9 ratio was added to each well. Subsequently, the cells were cultured at 37 °C for a duration of 3 h. Following the incubation period, the absorbance of each well was quantified at 450 nm using a microplate reader (Bio-Rad, CA, USA) to determine the level of cell viability or proliferation.

### In vitro migration and invasion assays

To evaluate the migration and invasion abilities of the cells, 24-well Transwells with 8 μm pore size (Corning, Inc., NY, USA) were utilized. In the top chamber, a total of 5 × 10^4^ MC38 cells in 200 µl of fresh medium (without FBS) were seeded. In the lower chamber, 600ul medium supernatant supplemented with a 20% concentration of FBS was added. For the invasion assay, the top chamber was coated with 200 mg/ml Matrigel (Corning, Inc., NY, USA) before adding 5 × 10^4^ cells. After a 48-hour incubation period, the cells that invaded through the Transwell membrane were stained and quantified. The migration assay was conducted without the use of Matrigel, following the same steps as the invasion assay.

### Immunofluorescence staining

Human tissue specimens were obtained from Xijing Hospital with the approval of the Institutional Review Board. CRC paired specimens were secured within 30 min post-tumor resection and preserved in paraformaldehyde for 48 h. Standard procedures were employed for dehydration and paraffin embedding. The specimens were treated with 3% H_2_O_2_ for 25 min to quench endogenous peroxidase activity. To block nonspecific binding, the tissue sections were pre-incubated with 10% normal goat serum for 30 min. Subsequently, they were incubated overnight at 4 °C with primary antibodies in a humidified chamber. The primary antibodies used to validate mast cells included mouse anti-human *CMA1* (AbCam, Cat# ab2377, 1:1000), mouse anti-human *TPSAB1* (Thermo Scientific, Cat#ab2378, 1:8000), rabbit anti-human *CPA3* (Sigma, Cat#HPA008689, 1:200), and rabbit anti-human *KIT* (AbCam, Cat#ab283653, 1:200), and rabbit anti-human *KITLG* (AbCam, Cat#ab52603, 1:200). Following thorough washing, the sections were mounted with an anti-fade reagent and covered with coverslips. Fluorescence images were captured using a NIKON ECLIPSE C1 microscope and further analysis was performed using CaseViewer software.

### CIBERSORT

In this study, we employed CIBERSORT [23], a computational tool, to analyze the cell type composition in bulk gene expression data. CIBERSORT estimates the relative abundance of different cell types within a sample based on the expression of specific gene markers, using a reference set of gene signatures. We utilized CIBERSORT to determine the proportion of resting and activated MCs in normal and CRC samples.

### CIBERSORTx

Cell composition deconvolution was conducted utilizing CIBERSORTx [24]. Our initial step was to generate a signature gene expression matrix using the CRC scRNA-seq dataset (GSE178341). We extracted raw count matrix data and cell type classifications from a subset of the Seurat object, which incorporated 1000 cells each from MC subsets, only including activated MCs and resting MCs. This raw count matrix was introduced into CIBERSORTx and subsequently normalized. The signature matrix, was established with CIBERSORTx, utilizing all genes to create the signature gene expression matrix. We evaluated the proportions of activated MCs and resting MCs in each CRC sample using CIBERSORTx, based on the bulk RNA-seq data (TCGA-CRC and GSE39582). To correct for cross-platform variation in the deconvolution of the RNA-seq data, we performed batch correction using S-mode with 100 permutations for significance analysis.

### Single-cell sequencing data processing

The three single-cell datasets used in this study underwent initial quality control by the original authors, and subsequent independent analyses were performed on each dataset. The expression of all cells was normalized using the “*LogNormalize*” function with a scale factor of 10,000. The top 2000 highly variable genes were selected based on their mean and dispersion, and regression of percent mitochondrial content was performed during scaling of these highly variable genes using the “*var.to.regress*” option. We zero-centered and scaled each gene to unit variance before principal component analysis (PCA) to minimize potential batch effects. Results were obtained through linear dimensionality reduction using PCA. The “*FindClusters*” function was utilized for preliminary clustering and annotation, employing 50 principal components with a resolution of 0.8. The UMAP method was used for nonlinear dimensionality reduction and visualization of cell clustering. Next, a second round of clustering was then performed to further characterize subpopulations of MCs.

### Expression difference analysis

To identify marker genes for each cluster or subset, we utilized the “*Findallmarkers*” function in *Seurat*. Genes were considered as differentially expressed genes (DEGs) of MCs in major cells if they met the following criteria: log fold-change of average expression > 1, pct.1 (percentage of expressed cells in MCs) > 0.7, pct.2 (percentage of expressed cells in other cells) < 0.3, and P value < 0.01. For MCs, genes were considered as upregulated DEGs in CRC if they met the following criteria: log fold-change of average expression > 0.25, pct.1 (percentage of expressed cells in MCs in CRC) > 0.25, and P value < 0.05. In analyzing expression differences between activated and resting MCs, we excluded 1,514 genes related to mitochondria, heat-shock proteins, ribosomes, and dissociation to eliminate noise and expression artifacts (Table S1).

### Trajectory analysis

To delineate the developmental trajectory of various MC subsets—including activated MCs, resting MCs, and proliferating MCs in the GSE178341 dataset—we employed the “monocle” package (version 2.28.0) [25]. The *DDRTree* method implemented with the “*reduceDimension*” function of Monocle 2 was used for dimensionality reduction and construction of pseudo-temporal order.

### Cell communication analysis

To investigate the interactions between MCs and other major cells, we utilized the Python-based software CellphoneDB [26]. Putative ligands and receptors were determined based on their expression on each cell. To accurately determine the extent of cell interactions, we performed a random sampling of 1,000 cells per population from the resting MCs, activated MCs, and major cell types in the GSE178341 dataset.

### Defining phenotype scores

To characterize the differences between various MC subsets, we obtained phenotype scores using the “*AddModuleScore*” function in the “*Seurat*” package. These scores were calculated based on the average expression of genes related to a particular phenotype.

In this study, the MC signature was defined by the average expression levels of five MC signature genes (*TPSAB1*, *TPSB2*, *CPA3*, *HPGDS*, and *MS4A2*). Additionally, the MC activation signature [27] and angiogenesis signature [28]were utilized to assess the characteristics of different MC subsets (Table S2).

### Spatial transcriptomic analysis

Standardization of the spatial transcriptomic data from the CRC sample was performed using the “*SCTransform*” function in the “*Seurat*” package. Dimensionality reduction and clustering were conducted using “*RunPCA*” and “*RunUMAP*” (with 15 principal components and a resolution of 0.8). Following the merging of similar clusters, we identified normal, stromal, and tumor regions in the CRC sample. To evaluate the spatial distribution of the MC activation signature, we utilized the “*AddModuleScore*” function in the “*Seurat*” package.

### Functional and pathway enrichment analysis

In this study, Metascape (http://metascape.org/) [29], a platform for gene function annotation analysis, was utilized to perform enrichment analysis of DEG of MCs between normal and CRC tissue, using Gene Ontology Biological Process (GO BP) gene sets.

Furthermore, the “*GSVA*” package (version 1.44.2) was used to perform gene set enrichment analysis (GSVA) between activated and resting MCs, utilizing Hallmark gene sets.

### Prognosis analysis of MC Signature and MC signature genes

To assess the prognostic role of MC signature in each cancer, we utilized univariate Cox regression and the Kaplan-Meier model. We analyzed four types of prognosis data, including overall survival (OS), disease-specific survival (DSS), disease-free interval (DFI), and progression-free interval (PFI). In the univariate Cox regression analysis, we used continuous expression data of MC signature. Furthermore, we performed Kaplan-Meier curve analysis using bivariate MC signature expression levels, with the cutoff determined by the “*surv-cutpoint*” function of the R package “*survminer*”. We presented the results as a heatmap, including log-rank p value, hazard ratio (HR) with 95% confidence interval (95%CI). To perform overall survival analysis of MC signature in two CRC cohorts, we utilized the “*survival*” package. Additionally, we conducted disease-free survival analysis of MC signature genes on the TCGA pan-cancer cohort using GEPIA2 [30].

### Statistical analysis

The statistical analysis in this study was conducted using R (version 4.2.2), GraphPad Prism (version 9), and Python (version 3.7). We utilized the Mann-Whitney U test to compare differences between two groups, and the Spearman method for correlation analysis. For survival analysis, we employed univariate Cox and Log-Rank methods, and a P-value of less than 0.05 considered statistically significant.

## Results

### Identification of MC signature genes and decreased MC density in CRC

**Fig. 1:**
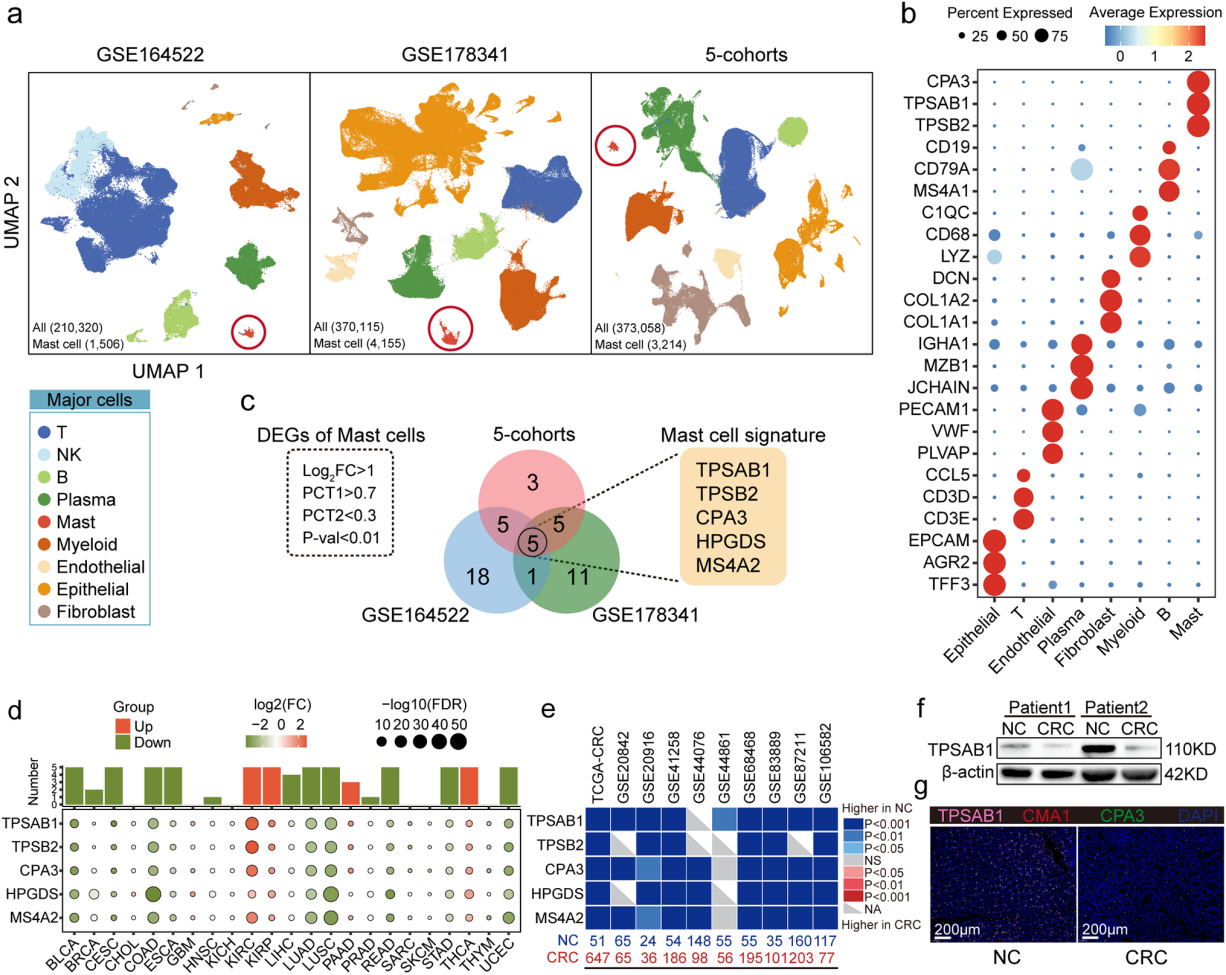
Identification of MC signature genes and decreased MC density in CRC. a. UMAP plots displaying the major cell types in the GSE164522 (n = 52 samples), GSE178341 (n = 100 samples), and 5-cohorts (n = 189 samples) datasets. b. Dot plots of marker genes for each major cell type in the 5-cohorts dataset. c. Venn diagrams (center) of differential expressed genes (DEGs) in MCs in the GSE164522, GSE178341, and 5-cohorts datasets, with the intersection showing the five MC signature genes (right). The criteria for screening DEGs are depicted within the dotted lines (left). d. Heatmap showing the expression of MC signature genes in each cancer (Normal vs. Tumor). Histogram shows the number of genes with statistical significance (upper). Red represents an increase in tumor expression, green represents a decrease in tumor expression, and only p-values < 0.05 are displayed. e. Heatmap showing the expression of the MC signature genes in 10 bulk RNA-seq cohorts of CRC (Normal vs. Tumor). Blue represents a decrease in tumor expression, dataset source (top), sample size (bottom). f. The expression of *TPSAB1* in human CRC tissue and paired NC tissue by Western blotting. g. Immunofluorescence staining of human CRC tissue and paired NC tissue. *TPSAB1* (pink), *CMA1* (green), *CPA3* (green), DAPI (blue), Bar, 200 μm. CRC, colorectal cancer; DEGs, differential expressed genes; FC, fold change; FDR, False Discovery Rate; MC, mast cell; NC, normal colorectum or adjacent colorectum

In this study, we analyzed three large CRC single-cell datasets (GSE164522, GSE178341, and 5-cohorts) comprising 341 samples and a total of 953,493 cells, identifying 8,875 MCs (Fig. 1a). We annotated major clusters based on defining marker genes and identified various cell types, including T cells (*CD3D*, *CD3E*), natural killer (NK) cells (*KLRF1*, *GNLY*), B cells (*CD19*, *MS4A1*), plasma cells (*MZB1*, *IGHA1*), MCs (*TPSAB1*, *TPSB2*, *CPA3*), myeloid cells (*LYZ*, *CD68*), endothelial cells (*PECAM1*, *VWF*), epithelial cells (*EPCAM*, *AGR2*), and fibroblasts (*DCN*, *COL1A2*) (Fig. 1a and b, Table S3).

To identify highly expressed genes that could serve as MC markers, we established strict criteria for differential gene screening, including a log2 fold change > 1 a proportion of expressed cells in MCs (PCT1) > 0.7, a proportion of expressed cells in other cell types (PCT2) < 0.3, and a P-value < 0.01. Five genes (*TPSAB1*, *TPSB2*, *CPA3*, *HPGDS*, and *MS4A2*) were identified as common DEGs across all three single-cell datasets and defined as MC signature genes (Fig. 1c).

The MC signature genes were used as MC markers to assess the density of MCs in bulk RNA-seq samples of both tumors and normal tissues. All five MC signature genes that achieved statistically significant (P < 0.05) were considered credible. Results showed that MC signature genes were significantly increased only in KICH, KIRC, and THCA but significantly decreased in BLCA, CESC, COAD, ESCA, LUAD, LUSC, READ, STAD, and UCEC (abbreviations of cancers are represented in Table S4), suggesting a reduction in MC density in the majority of tumors (Fig. 1d). Subsequent analyses in additional CRC cohorts verified a significant decrease in MC density in CRC (Fig. 1e). This was further corroborated by our Western Blot (Fig. 1f) and immunofluorescence (Fig. 1g) results derived from paired CRC and NC samples, which also pointed to a significant decrease in MC density within CRC.

In summary, we identified five reliable MC signature genes as markers, and our findings consistently demonstrated a reduction in MC density in CRC.

### Activation of MCs in CRC

**Fig. 2:**
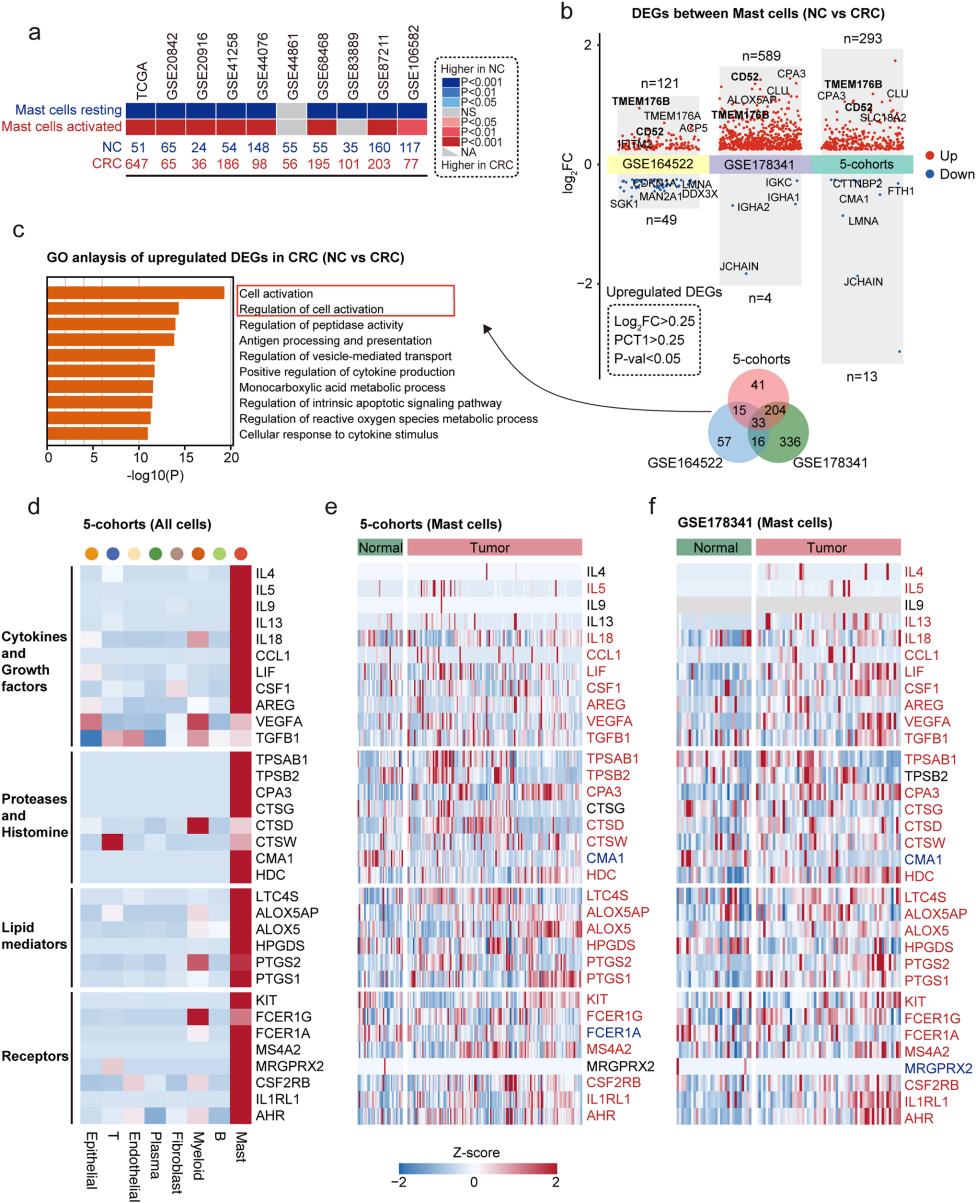
Activation of MCs in CRC. a. CIBERSORT-based analysis-generated heatmap showing the difference in the proportion of activated and resting MCs between NC and CRC in 10 bulk RNA-seq cohorts of CRC. Red indicates a higher proportion in CRC, while blue indicates a lower proportion in CRC. b. DEGs between NC MCs and CRC MCs from the GSE164522, GSE178341, and 5-cohort datasets (top). Venn diagram of upregulated DEGs in CRC MCs (bottom). Screening criteria are indicated by the dotted lines. c. Gene Ontology Biological Process (GO BP) enrichment analysis of upregulated DEGs in CRC MCs. d. Heatmap showing the expression of cytokine and growth factor, protease and histamine, lipid mediator, and various receptor-related genes across different major cell types (5-cohorts). e. Heatmap showing the expression of cytokine and growth factor, protease and histamine, lipid mediator, and various receptor-related genes in MCs (NC vs. CRC) (5-cohorts). The tissue type is indicated by the color above the heatmap. f. Heatmap showing the expression of cytokine and growth factor, protease and histamine, lipid mediator, and various receptor-related genes in MCs (NC vs. CRC) (GSE178341)

Activated MCs refer to MCs that have been stimulated by external factors, and they release biologically active substances, including histamine, cytokines, proteases, and lipid mediators such as leukotrienes and prostaglandins [6, 9]. Our research on the activation of MCs in CRC began with an unexpected finding. Using the CIBERSORT algorithm, we compared immune cell ratios between CRC and normal tissues in TCGA-CRC data and observed a significant increase in the proportion of activated MCs and a decrease in the proportion of resting MCs in CRC (Fig. 2a). This finding was further confirmed by data from nine additional CRC cohorts.

To gain a deeper understanding of the changes in MCs during the tumorgenesis of CRC, we compared gene expression differences between MCs in CRC tissue and normal tissue in three single-cell datasets (GSE164522, GSE178341, and 5-cohorts) (Fig. 2b and Table S5). Genes that met the criteria of log2 fold change > 0.25, a proportion of expressed cells in CRC MCs (PCT1) > 0.25, and a P-value < 0.05 were defined as DEGs. The number of DEGs enriched in MCs in CRC was significantly higher in all three datasets compared to those enriched in normal tissue, indicating a widespread gene expression increase in MCs during the progression of CRC. Notably, *TMEM176B* and *CD52* were among the top 5 significant genes in all three datasets, with *CD52* being reported as a marker of neoplastic MCs in patients with advanced systemic mastocytosis [31]. To study the functional changes in MCs during the progression from normal tissue to CRC, we used DEGs (n = 268) that were increased in two or more datasets for enrichment analysis. The results of the GO analysis showed that pathways related to cell activation were the most significantly enriched in MCs in CRC (Fig. 2c).

To better understand the activation features of MCs during CRC progression, this study analyzed the expression of receptor and mediator genes related to MCs in the 5-cohorts dataset (Fig. 2d). The results showed that MCs were the main population that expressed receptors for *IL-33* (*IL1RL1*) and *KIT*, with the highest expression levels of *MRGRPX2*, *CSF2RB*, and *AHR*. Notably, MCs were also the only cell population that expressed all three subunits of the high-affinity IgE receptor FcεR1 (i.e., *FCER1G*, *FCER1A*, and *MS4A2*). In addition, MCs showed high expression of signature proteases, including *TPSAB1*, *TPSB2*, *CPA3*, *CMA1*, and *CTSG*, with *CTSD* and *CTSW* being enriched in MCs but not limited to them. Moreover, MCs displayed high expression of genes involved in histamine biosynthesis (*HDC*), leukotriene biosynthesis (*LTC4S*, *ALOX5OP*, and *ALOX5*), and prostaglandin biosynthesis (*HPGDS*, *PTGS1*, *PTGS2*). MCs were the only cell population that expressed mRNA encoding diverse cytokines, chemokines, and growth factors, including *IL4*, *IL5*, *IL9*, *IL13*, *CCL1*, *LIF*, *CSF1*, and AREG. MCs also showed high expression of *IL18*, *VEGFA*, and *TGFB1*, although expression of these genes was not restricted to MCs.

The expression of MC receptors and mediators in CRC MCs and normal MCs was compared in the 5-cohorts (Fig. 2e) and GSE178341 (Fig. 2f) datasets. The results showed that most genes were significantly upregulated in CRC MCs, indicating that CRC MCs have a more activated MC phenotype compared to normal MCs. The exception was *CMA1*, which encodes chymase and was significantly more highly expressed in normal tissue.

In summary, the evidences indicate activation of MCs in CRC.

### Heterogeneity of MCs in CRC

**Fig. 3:**
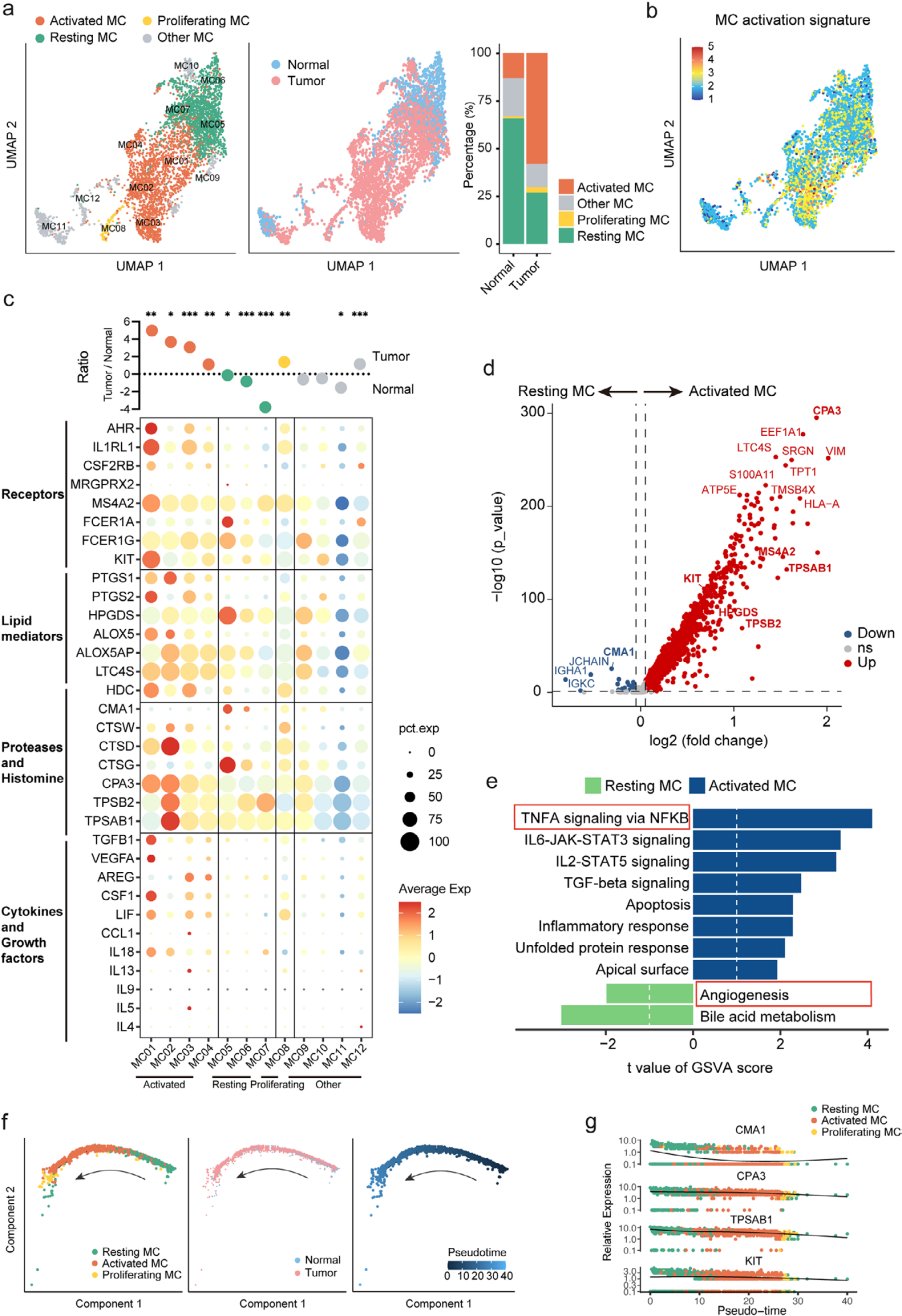
Heterogeneity of MCs in CRC. a. UMAP plots of 4,155 MCs colored by cluster (left) and tissue type (center) in the GSE178341 dataset. Bar charts show the proportion of MC subtypes in different tissues (right). b. UMAP plots of MC activated signature. c. Log ratio of average fraction per MC clusters in tumor to normal tissue (top). Mann-Whitney U test, *: p < 0.05, **: p < 0.01, ***: p < 0.001. Dot plots of cytokine and growth factor, protease and histamine, lipid mediator and various receptor-related gene expression in MC clusters (bottom). d. Volcano plot of differentially expressed genes between resting and activated MCs. e. Differential pathway enriched in resting and activated MCs by GSVA, showing the top 10 significant enriched hallmark terms. f. Differentiation trajectory of MCs with each color coded for MC subsets (left), tissue types (center), and pseudotime (right). g. Pseudotime trajectory of *CMA*1, *CPA3*, *TPSAB1*, and *KIT* expression levels

The advent of single-cell analysis has enabled the characterization of MC activation during CRC from the perspective of MC heterogeneity. In the GSE178341 cohort, analysis of 4,155 MCs led to the identification of 12 clusters corresponding to 4 distinct MC subtypes (Fig. S1a). The MC11 and MC12 clusters, enriched in *MZB1* and *CD3D* respectively, were considered B cell doublets and T cell doublets, respectively, while the MC09 and MC10 clusters, enriched in interferon-related genes (IFITs) and mitochondrial genes (MTs), respectively, were grouped as “Other MCs”. The MC08 cluster, enriched in genes related to proliferation such as *MKI67*, was named as “proliferation MCs”. Based on the overall expression levels of MC receptor and mediator genes, the relatively high MC01-04 clusters were named “activated MCs”, while the MC05-07 clusters were named “resting MCs” (Fig. 3a and Fig. S1a). The significant enrichment of the MC activation signature in activated MCs compared to resting MCs further supported the naming of these MC clusters (Fig. 3b). The proportion of activated MCs in CRC was significantly higher, while the proportion of resting MCs was significantly higher in normal tissue (Fig. 3a and c), indicating that the activation of MCs in CRC is due to a higher proportion of activated MCs.

In the comparison of activated MCs and resting MCs, it was found that most MC receptor and mediator-related genes, including the five major MC signatures (*TPSAB1*, *TPSB2*, *CPA3*, *HPGDS*, and *HS4A2*), were enriched in activated MCs, while *CMA1* was enriched in resting MCs (Fig. 3d and Table S6). GSVA analysis revealed that the TNFA signature via NF-κB was most significantly enriched in activated MCs, whereas the angiogenesis-related pathway was enriched in resting MCs (Fig. 3e). This finding was supported by the result of the angiogenesis score, which confirmed the higher angiogenesis feature of resting MCs compared to activated MCs (Fig. S1b). Additionally, the enrichment of MHC-I and MHC-II related genes in activated MCs indicated that activated MCs have a stronger antigen-presenting function (Fig. S1c). The heterogeneity observed in MCs within CRC was also corroborated in the 5-cohorts dataset (Fig. S2a-d).

In addition, our pseudo-temporal analysis using Monocle 2 further supported a transition from resting to activated MCs (Fig. 3f), during which we also observed a decline in *CMA1* expression (Fig. 3g).

### High MC signature associated with favorable outcome in tumors

**Fig. 4:**
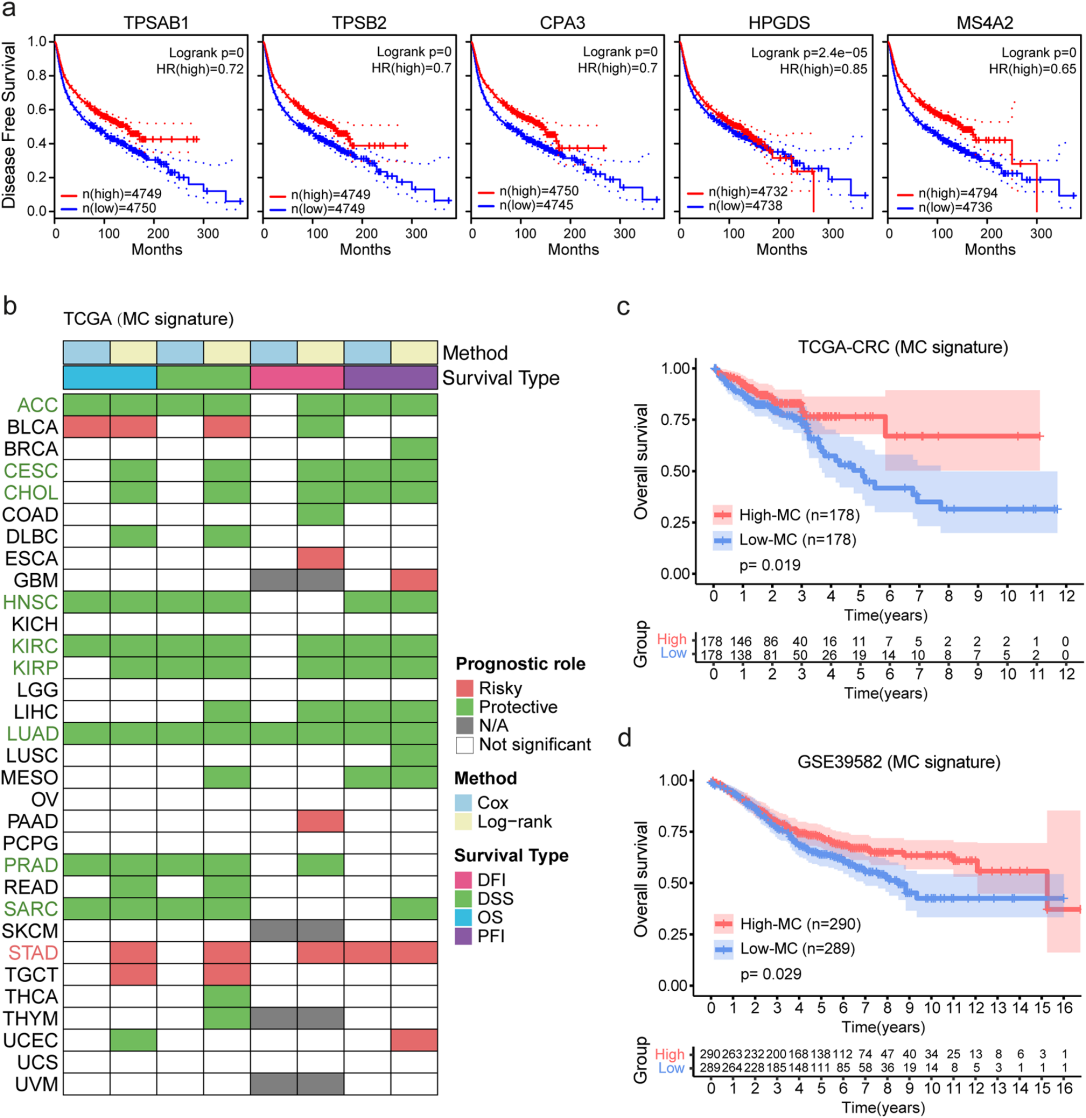
MC signature predicts better prognosis. a. Kaplan-Meier disease-free survival curves grouped by MC signature genes (*TPSAB1*, *TPSB2*, *CPA3*, *HPGDS*, and *MS4A2*) in pan-cancer. b. Summary of the correlation between the expression of MC signature and overall survival (OS), disease-specific survival (DSS), disease-free interval (DFI), and progression-free interval (PFI) based on univariate Cox regression and Kaplan-Meier models. Red indicates factors that are detrimental to the prognosis of cancer patients, while green represents protective factors. Only *p*-values < 0.05 are displayed. c. The Kaplan-Meier overall survival curve of the MC signature in TCGA-CRC is shown, with the High-MC group and Low-MC group including patients with CRC who had MC signature expression in the top 30% and bottom 30%, respectively. d. Kaplan-Meier overall survival curve of MC signature in GSE39582

The impact of MCs on cancer prognosis remains controversial [9–11]. Based on the Kaplan-Meier model of TCGA pan-cancer data, we found that high expression of five MC signature genes was associated with better disease-free survival (Fig. 4a). In addition, we used eight prognostic indicators based on univariate Cox regression and Kaplan-Meier models for OS, DSS, DFI, and PFI to evaluate the impact of MC signature on prognosis in different cancers. A reliable result was considered if statistical significance (*p* < 0.05) was reached in at least four indicators. The results showed that the MC signature had a significant protective effect in 10 cancer types, including ACC, CESC, CHOL, HNSC, KIRC, KIRP, LIHC, LUAD, PRAD, and SARC, but was only associated with poor prognosis in STAD (Fig. 4b). Kaplan-Meier analysis of the TCGA-CRC (log-rank, *p* = 0.019) and GSE39582 (log-rank, *p* = 0.029) also indicated that a high MC signature was associated with better overall survival in CRC cohorts (Fig. 4c and d, Fig. S3a, and Fig. S3b).

Additionally, we employed CIBERSORTx to delve into the influence of MC phenotypes on prognosis and observed a heightened proportion of activated MCs in CRC samples compared to normal tissues, concomitant with a reduction in resting MCs (Fig. S3c). However, no statistically significant differences were discovered concerning the proportions of activated and resting MCs calculated via CIBERSORTx in relation to CRC prognosis (Fig. S3d and Fig. S3e).

These findings demonstrate the important role of MCs in the prognosis of CRC patients and their potential as a protective prognostic biomarker.

### *KITLG/KIT* signaling in MC activation and CRC inhibition

**Fig. 5:**
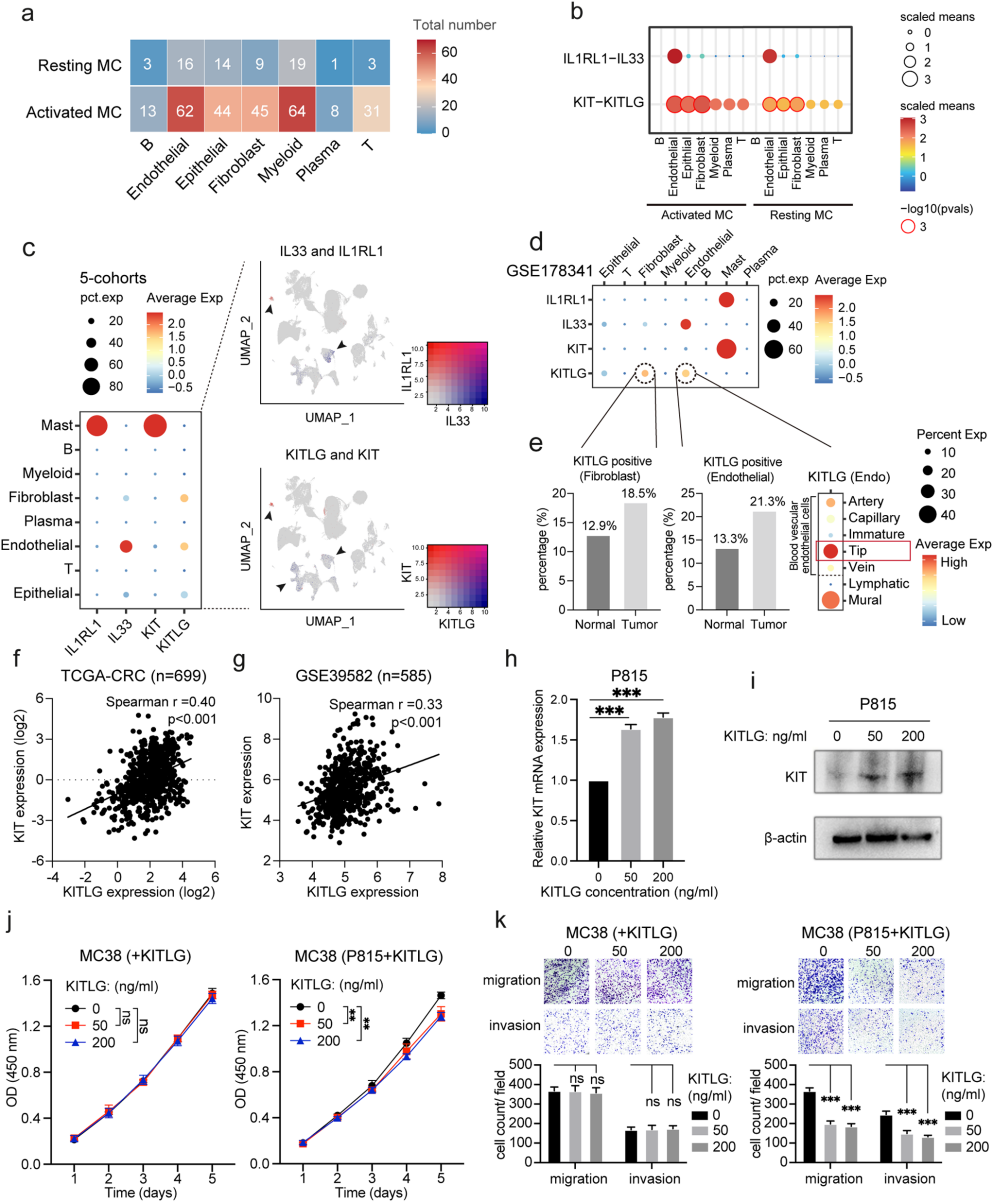
*KITLG/KIT* signaling in MC activation and CRC inhibition. a. Heatmap generated by CellphoneDB analysis showing the potential ligand-receptor interactions between resting and activated MCs and other major cell types in CRC (GSE178341). Numbers indicate the number of potential ligand-receptor pairs. b. Dot plots of interactions between resting and activated MCs and other major cell types along the *IL33*-*IL1RL1* and *KITLG*-*KIT* axes. c. Dot plots displaying the expression of *IL1RL1*, *IL33*, *KIT*, and *KITLG* in different major cell types (5-cohorts) (left), and UMAP plots displaying the expression of *IL33* and *IL1RL1* (right upper), and *KITLG* and *KIT* (right bottom). d. Dot plots showing the expression of *IL1RL1*, *IL33*, *KIT*, and *KITLG* in different major cell types (GSE178341). e. Bar plots comparing the positive rate of *KITLG* expression between normal tissue and CRC in fibroblasts (left) and endothelial cells (center). Dot plots showing the expression of *KITLG* in different endothelial cell subsets (right). f. Correlation analysis of *KITLG* and *KIT* expression in TCGA-CRC (Spearman test). g. Correlation analysis of *KITLG* and *KIT* expression in GSE39582 (Spearman test). h. qRT-PCR analysis shows *KIT* mRNA expression level in P815 after manipulating different concentrations of *KITLG* protein. i. Western blot analysis shows *KIT* protein expression level in P815 after manipulating different concentrations of *KITLG* protein. j. CCK-8 assay comparing the proliferative capacity of CRC cells when exposed to medium with only different *KITLG* concentrations (left), compared to medium from p815 coculture with varying *KITLG* concentrations (right). Optical density (OD) was monitored daily for a 5-day period. k. Transwell analysis showing the impact on CRC cell migration and invasion when exposed to medium with only different *KITLG* concentrations (left), compared to medium from p815 coculture with varying *KITLG* concentrations (right). All data are shown as the mean ± SD. **: p < 0.01, ***: p < 0.001

In this section, we aimed to identify the potential causes of MC activation in the CRC TME. Using the GSE178341 dataset, we performed CellphoneDB analysis and found that activated MCs had a higher number of interactions with other cell types compared to resting MCs. The prediction results also showed that activated MCs had the highest number of interacting receptors with myeloid cells, endothelial cells, fibroblasts, and epithelial cells (Fig. 5a).

Previous studies have shown that *KITLG* (*SCF*, stem cell factor) [32–34] and *IL33* [27, 35, 36] are key molecules that can promote MC activation by binding to the surface receptor *KIT* and *IL1RL1*, respectively. To investigate the relationship between *IL33*-*IL1RL1* and *KITLG* -*KIT* axes with MC activation, we conducted a CellphoneDB analysis and found that the average expression of the *KITLG* -*KIT* axis interacting with upstream cells was significantly higher in activated MCs compared to resting MCs (Fig. 5b). Furthermore, single-cell analysis identified the upstream cell types expressing *IL1RL1* and *KITLG*. *IL1RL1* was found to be almost exclusively expressed in endothelial cells (Fig. 5c and d), while *KITLG* was mainly highly expressed in endothelial cells and fibroblasts, followed by epithelial cells.

We compared the expression of *KITLG* in CRC and normal tissues and found that the expression of *KITLG* in fibroblast cells in CRC was significantly higher than that in normal tissues (*p* = 5.45E-12) (Fig. 5e) (Table S7). Similarly, the expression of *KITLG* in endothelial cells in CRC was also significantly higher than that in normal tissues (*p* = 1.97E-20). Further sub-group analysis of endothelial cells revealed that *KITLG* expression is enriched in tip cells, a subset marked by *ESM1* and *PGF* and significantly elevated in CRC (Fig. 5e) (Table S8) [37]. Consistent with a recent study that reported higher *KITLG* expression in ACTA2 + vascular smooth muscle cells (VSMCs) compared to other stromal cell types [38], our findings also indicate that *KITLG* expression is enriched in mural cells, which are marked by *RGS5* and *ACTA2* (Fig. 5e). Moreover, a significant positive correlation between *KITLG* and *KIT* expression was observed in both the TCGA-CRC (Fig. 5f) and GSE39582 cohorts (Fig. 5g). These findings suggest that the increased expression of *KITLG* in endothelial cells and fibroblasts in CRC may be a significant cause of MC activation.

We further explored the effects of *KITLG/KIT* pathway activation in MCs on the proliferation and migration of CRC cells through in vitro experiments. Initially, PCR and Western Blot analyses were performed to assess *KIT* expression in the P815 cell line after 48 h of co-culturing with *KITLG*. Both assays confirmed an elevated *KIT* expression in the P815 cells (Fig. 5h and i), validating the successful activation of the *KITLG/KIT* signaling pathway in MCs. Subsequent CCK8 (Fig. 5j) and Transwell assays (Fig. 5k) revealed that as the concentration of *KITLG* increased, the presence of *KITLG*-activated P815 cells led to a significant reduction in both the proliferation and migration of CRC cells. In contrast, adding *KITLG* alone in the medium of MC38 cell line did not produce any notable changes in CRC cell behavior (Fig. 5j and k). These results suggest that *KITLG* can inhibit CRC cell proliferation and migration through its action on MCs.

In summary, our findings indicate that the *KITLG/KIT* signaling pathway may be a key mechanism for activating MCs, which in turn inhibits the proliferation and migration of CRC cells.

### Spatial colocalization of MCs with fibroblasts and endothelial cells

**Fig. 6:**
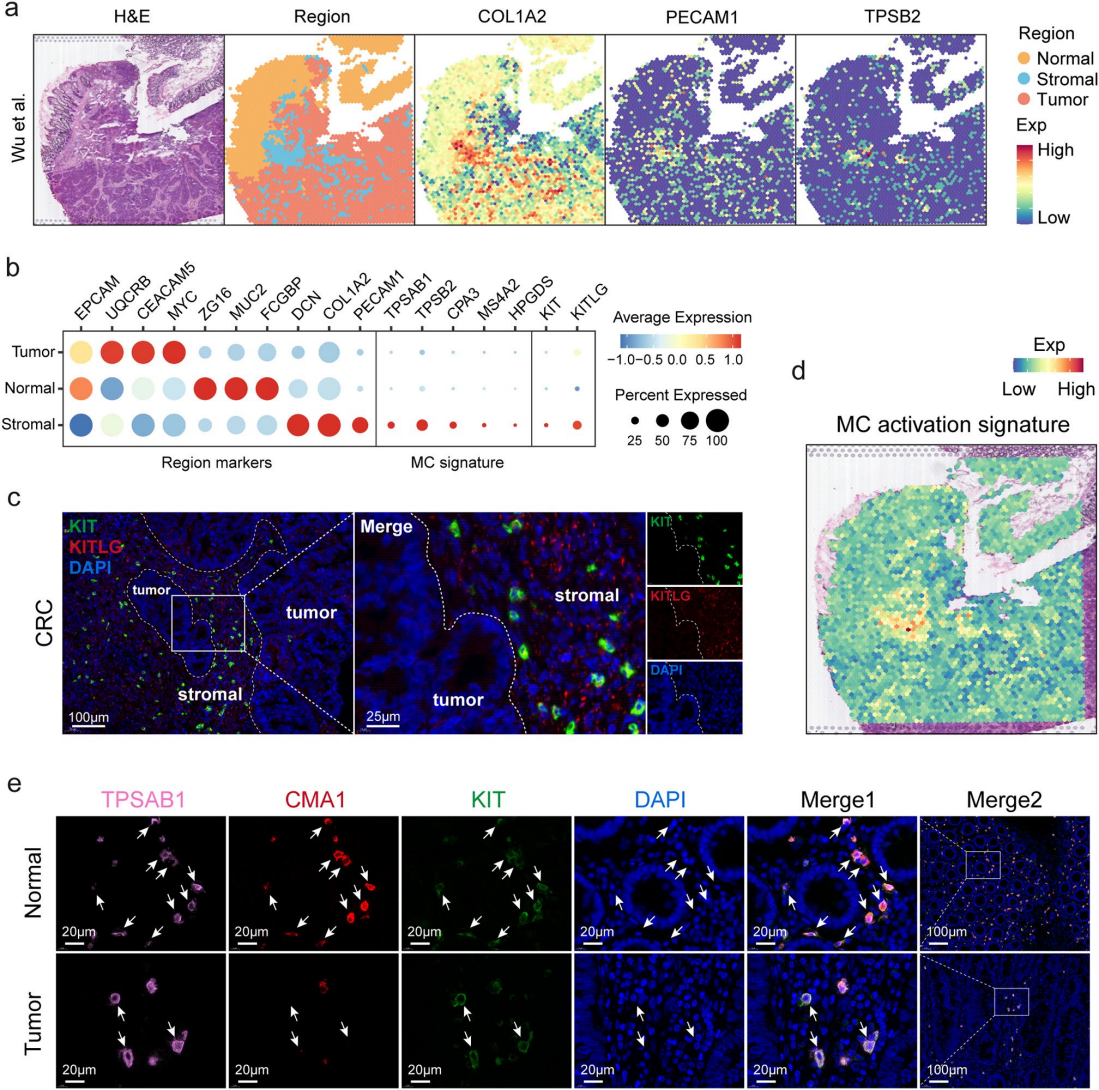
Spatial co-localization of MCs with fibroblasts and endothelial cells. a. Spatial plots of H&E staining (column 1), tissue regions (column 2), fibroblasts (COL1A2) (column 3), endothelial cells (PECAM1) (column 4), and MCs (TPSB2) (column 5) in a CRC sample. b. Dot plots display the expression of the regional marker genes (left), MC signature genes (middle), and expression of *KIT* and *KITLG* (right) in different tissue regions. c. Immunofluorescence staining of human CRC tissue. *KITLG* (red), *KIT* (green), DAPI (blue). Dotted lines demarcate the boundary between the tumor and the stromal regions. d. Spatial visualization of the MC activation signature in the CRC sample. e. Immunofluorescence staining of human CRC tissue and paired NC tissue. *TPSAB1* (pink), *CMA1* (red), *KIT* (green), DAPI (blue), in individual and merged channels are shown

To explore the relationship between the spatial distribution of MCs and their activation, we analyzed the spatial distribution of MCs in CRC tissue using spatial transcriptomics data. Based on transcriptome expression and HE tissue information, 3,138 spots were divided into three regions: normal region, stromal region, and tumor region (Fig. 6a). The stromal region is enriched with fibroblasts (*DCN*, *COL1A2*) and endothelial cells (*PECAM1*), and the five MC signature genes and *KIT*, *KITLG* are also significantly enriched in the stromal region, indicating that MCs co-localize spatially with fibroblasts and endothelial cells (Fig. 6b).

Further investigations revealed an additional spatial co-localization of MCs with mural cells (identified by *RGS5* and *ACTA2*) within the stromal region (Fig. S4a-c). We validated these transcriptomic insights with immunofluorescence assays, specifically confirming the co-localization of *KIT* and *KITLG* proteins within the stromal compartment of CRC tissues (Fig. 6c). In addition, the MC activation signature also showed enrichment in the stromal region (Fig. 6d), suggesting that the stromal environment may serve as a critical niche for MC activation in CRC.

Lastly, our immunofluorescence results from the paired CRC and NC samples revealed that MCs were uniformly distributed within the stromal region interspersed among the endothelial glandular structures (Fig. 6e). Crucially, we observed a significant reduction in the expression of *CMA1* in individual MCs within CRC tissues, along with an increase in *TPSAB1* and *KIT*, compared to the NC paired samples (Fig. 6e). This confirmed the shift from a resting to an activated phenotype in MCs during tumor formation.

## Discussion

This study utilized large-scale single-cell datasets to investigate the heterogeneity of MCs in CRC. The most significant finding was the observed activation of MCs in CRC. Further investigation revealed that the upregulation of *KITLG* expression in endothelial cells and fibroblasts in CRC may play a role in the activation of MCs, ultimately exerting a protective effect on CRC patients. By providing new insights into the heterogeneity and role of MCs in CRC, this study advances our understanding of the complex interplay between MCs and tumors and underscores the potential for MCs as a therapeutic target in cancer.

MC heterogeneity was initially described in the mid-1960s based on differences in histochemical staining features, leading to the development of the concept of connective tissue MCs (CTMCs) and mucosal MCs (MMCs) [39]. In humans, different MC subpopulations have been defined by their protease content; those that express tryptase (MC_T_), tryptase and chymase (MC_TC_), and chymase only (MC_C_) [40–42]. Recently, MCs have been classified into two or more subtypes according to their different roles in tumors, including pro-tumorigenic MCs and anti-tumorigenic MCs [43, 44]. In this study, we defined activated MCs based on single-cell transcriptomic features as those with high expression of receptors and MC mediators, while resting MCs have low expression. We observed that the proportion of activated MCs was higher in CRC compared to normal tissues, while the proportion of resting MCs was lower, providing insights into the activation of MCs in CRC from the perspective of MC heterogeneity.

It is commonly believed that MCs accumulate in tumors, including CRC [44–46]. However, recent evidence suggests a decrease in MCs during CRC progression [15, 18]. In this study, we used a single-cell-based MC signature to evaluate the density of MCs in normal and tumor tissues from bulk RNA-seq data. Our results showed a significant decrease in MC density in the majority of tumors, with a particularly notable decrease in MC density in CRC. This finding supports the growing evidence of decreased MC density during CRC progression. Furthermore, the failure of previous studies to detect MC activation in tumors may also be related to the decrease in MC density in tumors.

The role of MCs in tumors remains a topic of debate [6, 9–11, 42, 43, 47, 48]. Recently, a single-cell study on MCs in pan-cancer classified tumor MCs into two subtypes: anti-tumorigenic MCs, which are characterized by a high *TNF*/*VEGFA* expression ratio, and pro-tumorigenic MCs, which are characterized by a low *TNF*/*VEGFA* expression ratio [44]. In our study, we found that both anti- and pro-tumorigenic functions of activated MCs were enhanced by the general upregulation of gene expression, such as increased expression of the anti-tumorigenic gene *TNF* and the pro-tumorigenic gene *VEGFA*. Additionally, MCs can also function as antigen-presenting cells (APCs) for T cells [49, 50]. The higher expression of MHC-I and MHC-II-related genes in activated MCs compared to resting MCs suggests that activated MCs may also play a role as APCs in anti-tumor immunity. Angiogenesis is considered a key factor in MC-mediated tumorigenesis [6, 11, 43], and interestingly, our study found that resting MCs exhibit more angiogenic features, which may be related to the higher expression of the chymase gene *CMA1* in resting MCs [51]. Taking these findings into consideration, we tend to believe that activated MCs in CRC play a more protective role against tumorigenesis. Furthermore, both our in vitro experimental findings and prognostic analyses consistently support the protective role of activated MCs in CRC patients.

*KITLG* is a cytokine that can act as both a transmembrane and soluble protein [52] and plays a critical role in activating MCs by binding to its receptor, *KIT*. The *KITLG*/*KIT* (*SCF*/*c-Kit*) axis is widely recognized as a critical pathway for MC activation [32–34]. Our single-cell analysis of CRC samples revealed that *KITLG* enrichment is present in fibroblasts and endothelial cells. The results from TCGA-CRC showed a positive correlation between *KITLG* and *KIT* expression, which is further corroborated by our in vitro experiments demonstrating that *KITLG* protein can promote *KIT* expression in MCs. Furthermore, we found that *KITLG* expression was significantly higher in fibroblasts and endothelial cells in CRC samples compared to normal tissues. These findings suggest that *KITLG* expression in fibroblasts and endothelial cells could be a critical upstream factor in the activation of MCs in CRC. These results provide new insights into the molecular mechanisms underlying MC activation in CRC and highlight the potential of the *KITLG*/*KIT* axis as a target for cancer therapy. For instance, elevating *KITLG* expression in fibroblasts and endothelial cells could stimulate MC activation and proliferation through the *KITLG/KIT* pathway, thereby inhibiting tumor cells and enhancing patient outcomes.

Mature MCs are known to strategically distribute themselves in close proximity to blood and lymphatic vessels as well as nerves, enabling them to promptly respond to pathogens and other foreign substances [53–55]. Our spatial transcriptome analysis and immunofluorescence results also revealed the co-localization of MCs with fibroblasts and endothelial cells in the stromal region. Furthermore, the significant enrichment of the *KITLG*-*KIT* axis and MC activation signature in the stroma further emphasizes the stromal region as a crucial site for MC activation.

Two limitations of this study are worth mentioning. Firstly, the definition of activated MCs in this study is rough and may not fully capture the diversity of activated MC subpopulations. For instance, MC01 has high *KIT* expression but low *TPSAB1* expression, while MC02 has high *TPSAB1* expression but low *KIT* expression. Secondly, the mechanisms underlying the upregulation of *KITLG* expression in fibroblasts and endothelial cells in CRC are not yet fully understood. Further research is necessary to clarify these mechanisms and explore other potential upstream factors that may contribute to MC activation in CRC.

**Fig. 7:**
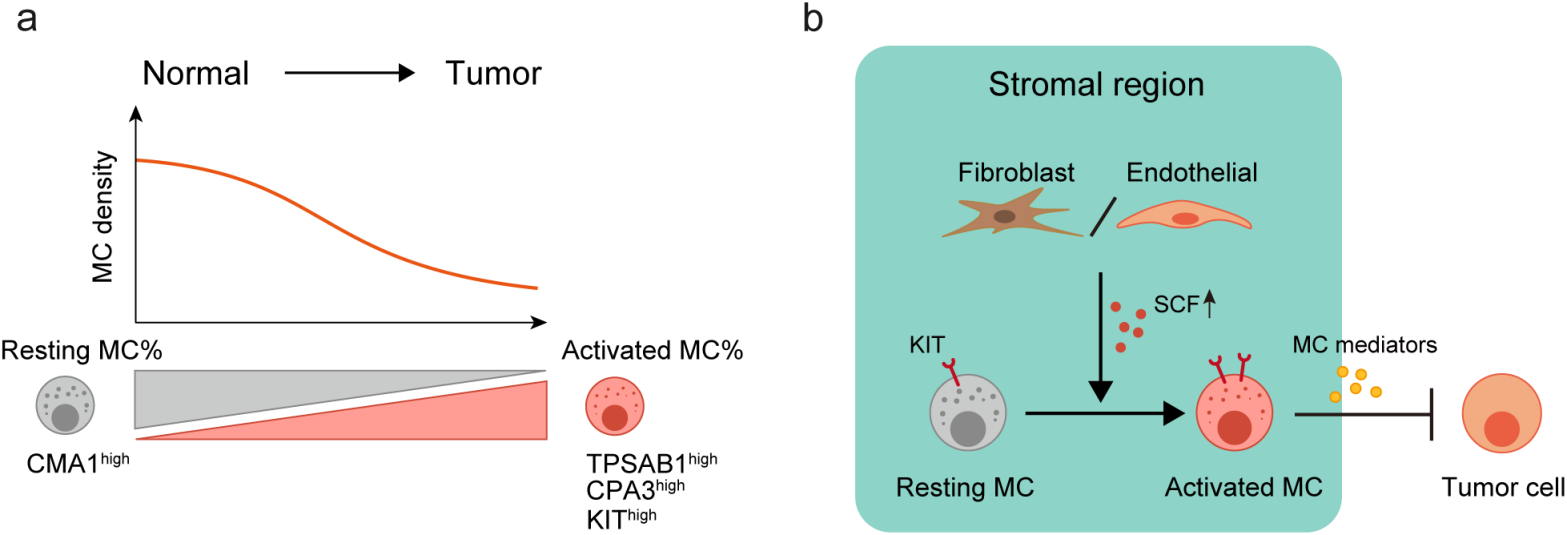
Diagram of MC Activation in CRC. a. Compared to normal tissue, the overall density of MCs decreases in CRC, but the phenotype of MCs changes from resting MCs with high *CMA1* expression to activated MCs with high expression of *TPSAB1*, *CPA3*, and *KIT*. b. *KITLG* (SCF) expressed by fibroblasts and endothelial cells in the stromal region increases in the TME, which may promote MC activation through the *KITLG*-*KIT* axis and thereby suppress tumor progression

In conclusion, our study demonstrated a decrease in MC density in CRC compared to normal tissues, but a shift in MC phenotype from *CMA1*^high^ resting cells to activated *TPSAB1*^high^, *CPA3*^high^, and *KIT*^high^ cells (Fig. 7a). We also identified that elevated expression of *KITLG* (*SCF*) in the TME by fibroblasts and endothelial cells may activate MCs via the *KITLG*-*KIT* axis, potentially inhibiting tumor progression (Fig. 7b). By redefining the heterogeneity of MCs in CRC at the single-cell level, our study has the potential to provide valuable insights for developing effective immunotherapy targeting MCs for CRC.

### Electronic supplementary material

Below is the link to the electronic supplementary material.

**Supplementary Material 1**: **Figure S1**. Heterogeneity of MCs and Their Functional Characteristics. **(a)**. Dot plot displaying the clustering and marker gene expression of MCs based on cluster (GSE178341). **(b)**. Violin plot comparing the differences in angiogenesis signature between resting and activated MCs. **(c)**. Dot plot comparing the expression of MHC-I and MHC-II related genes between resting and activated MCs

**Supplementary Material 2**: **Figure S2**. Heterogeneity of MCs in CRC (5-cohorts). **(a)**. Log ratio of average fraction per MC clusters in tumor to normal tissue (top). Mann-Whitney U test, *: p < 0.05, **: p < 0.01, ***: p < 0.001. Dot plots of cytokine and growth factor, protease and histamine, lipid mediator and various receptor-related gene expression in MC clusters (bottom). **(b)**. Dot plot displaying the clustering and marker gene expression of MCs based on cluster (5-cohorts). **(c)**. UMAP plots of 3,214 MCs colored by cluster (left) and tissue type (right) in the 5-cohorts dataset. Bar charts show the proportion of MC subsets in different tissues (bottom). **(d)**. Volcano plot of differentially expressed genes between resting and activated MCs

**Supplementary Material 3**: **Figure S3**. Supplementary Prognosis Information. **(a)**. Kaplan-Meier overall survival curve of MC signature in TCGA-COAD. **(b)**. Kaplan-Meier overall survival curve of MC signature in TCGA-READ. **(c)**. Comparison of activated MC fraction (left) and resting MC fraction (right) between tumor and normal tissue in TCGA-CRC. Mann-Whitney U test, ****: p < 0.0001. **(d)**. Kaplan-Meier overall survival curve of activated MC fraction in TCGA-CRC. **(e)**. Kaplan-Meier overall survival curve of activated MC fraction in GSE39582. All Kaplan-Meier curves above were generated using median values for grouping. The activated MC fraction and resting MC fraction in c-e were based on results obtained using CIBERSORTx

**Supplementary Material 4**: **Figure S4**. Spatial co-localization of MCs with mural cells in stromal region. **(a)**. Spatial plots of tissue regions, MCs (*TPSAB1*), tip cells (*PGF* and *ESM1*), and mural cells (*RGS5* and *ACTA2*) in a CRC sample. **(b)**. Dot plots display the expression of MC marker gene, tip cell marker genes, and mural cell marker genes in different tissue regions. **(c)**. Comparison of *PGF*, *ESM1*, *RGS5*, and *ACTA2* expressions between *TPSAB1*- and *TPSAB1*+ spatial plots in stromal region. Mann-Whitney U test, **: p < 0.01, ****: p < 0.0001

**Supplementary Material 5**: **Table S1**. 1514 genes associated with mitochondria, heat-shock proteins, ribosome, and dissociation. **Table S2**. Gene lists of MC signature, MC activation signature, and angiogenesis signature. **Table S3**. DEGs between major cells. **Table S4**. Abbreviations. **Table S5**. DEGs between MCs (NC vs. CRC). **Table S6**. DEGs between MCs in GSE178341 (Resting MC vs. Activated MC). **Table S7**. DEGs of fibroblasts and endothelial cells between NC and CRC. **Table S8**. DEGs between endothelial cell subsets

## Data Availability

The data used in this study are all from public datasets, which are detailed in the Methods section of the manuscript. The codes used in this study are available on Github (https://github.com/LPC19970117/Mast-cell).
