# Supplementary figures and images for: Single-cell analysis unveils activation of mast cells in colorectal cancer microenvironment
Source: Cell Biosci. 2023 Nov 29;13:217. doi: 10.1186/s13578-023-01144-x (PMC10687892; doi:10.1186/s13578-023-01144-x)

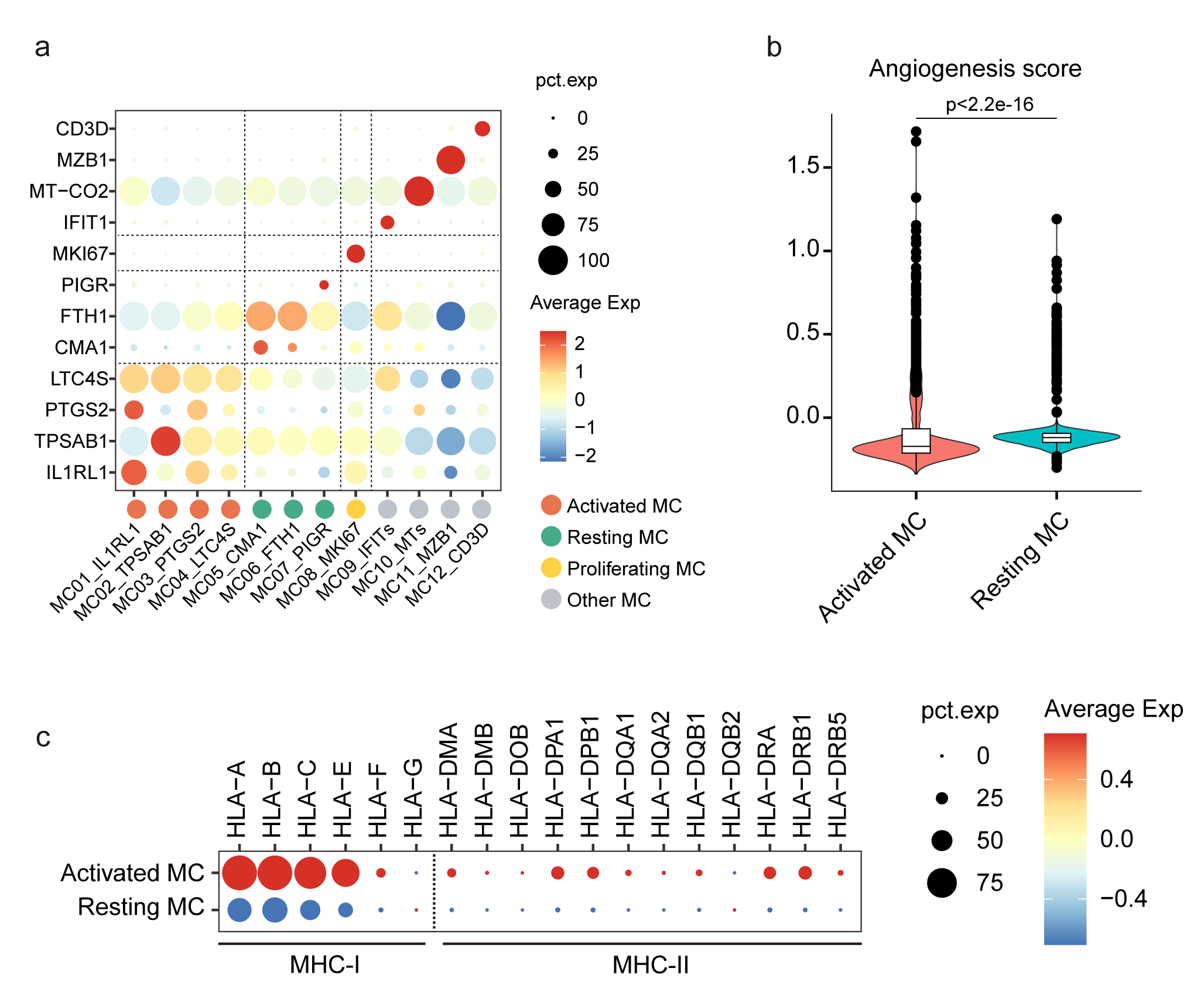

Supplement: Supplementary file 1 — Supplementary Material 1: Figure S1. Heterogeneity of MCs and Their Functional Characteristics. (a). Dot plot displaying the clustering and marker gene expression of MCs based on cluster (GSE178341). (b). Violin plot comparing the differences in angiogenesis signature between resting and activated MCs. (c). Dot plot comparing the expression of MHC-I and MHC-II related genes between resting and activated MCs [file 13578_2023_1144_MOESM1_ESM.tif]

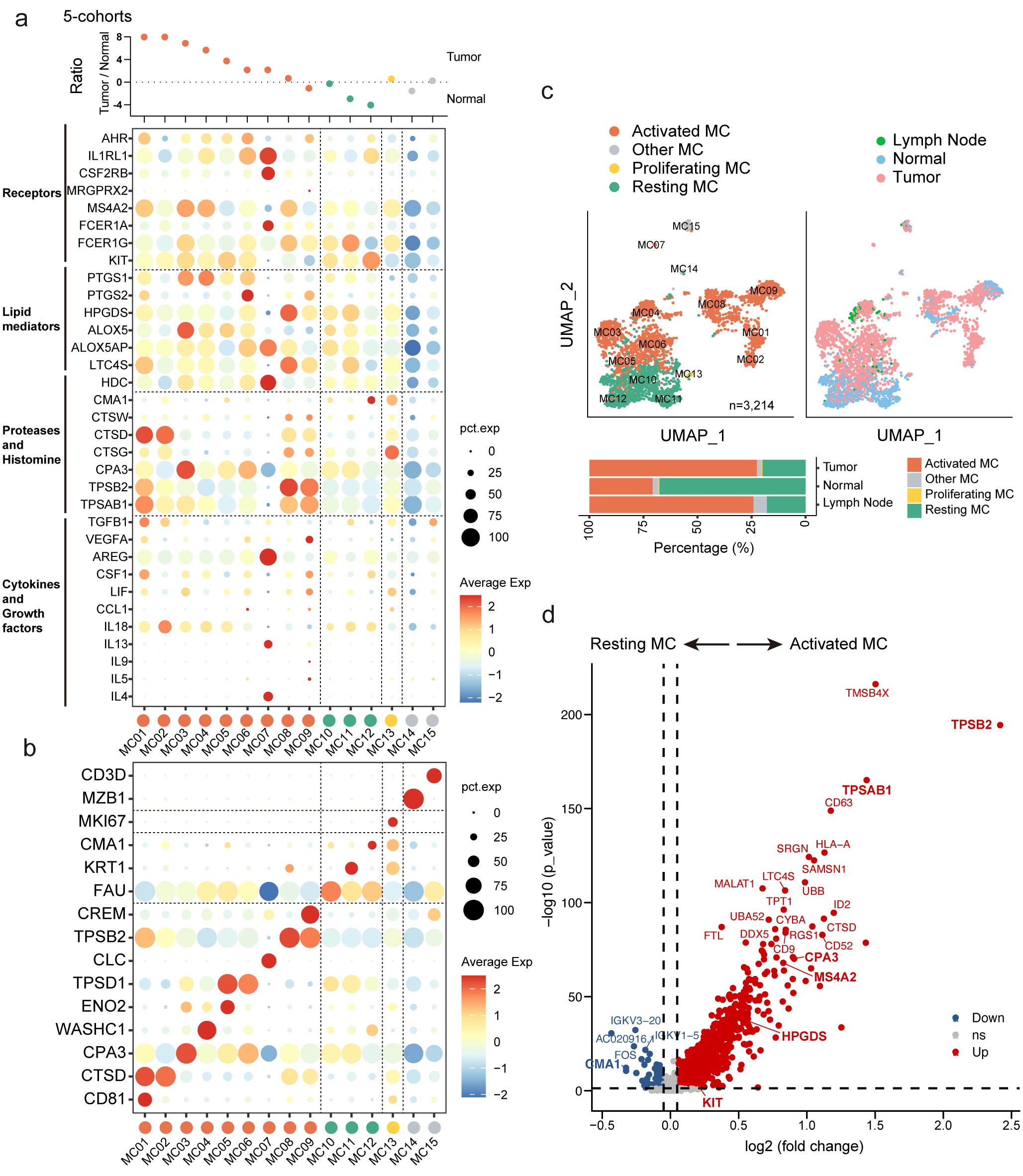

Supplement: Supplementary file 2 — Supplementary Material 2: Figure S2. Heterogeneity of MCs in CRC (5-cohorts). (a). Log ratio of average fraction per MC clusters in tumor to normal tissue (top). Mann-Whitney U test, *: p < 0.05, **: p < 0.01, ***: p < 0.001. Dot plots of cytokine and growth factor, protease and histamine, lipid mediator and various receptor-related gene expression in MC clusters (bottom). (b). Dot plot displaying the clustering and marker gene expression of MCs based on cluster (5-cohorts). (c). UMAP plots of 3,214 MCs colored by cluster (left) and tissue type (right) in the 5-cohorts dataset. Bar charts show the proportion of MC subsets in different tissues (bottom). (d). Volcano plot of differentially expressed genes between resting and activated MCs [file 13578_2023_1144_MOESM2_ESM.tif]

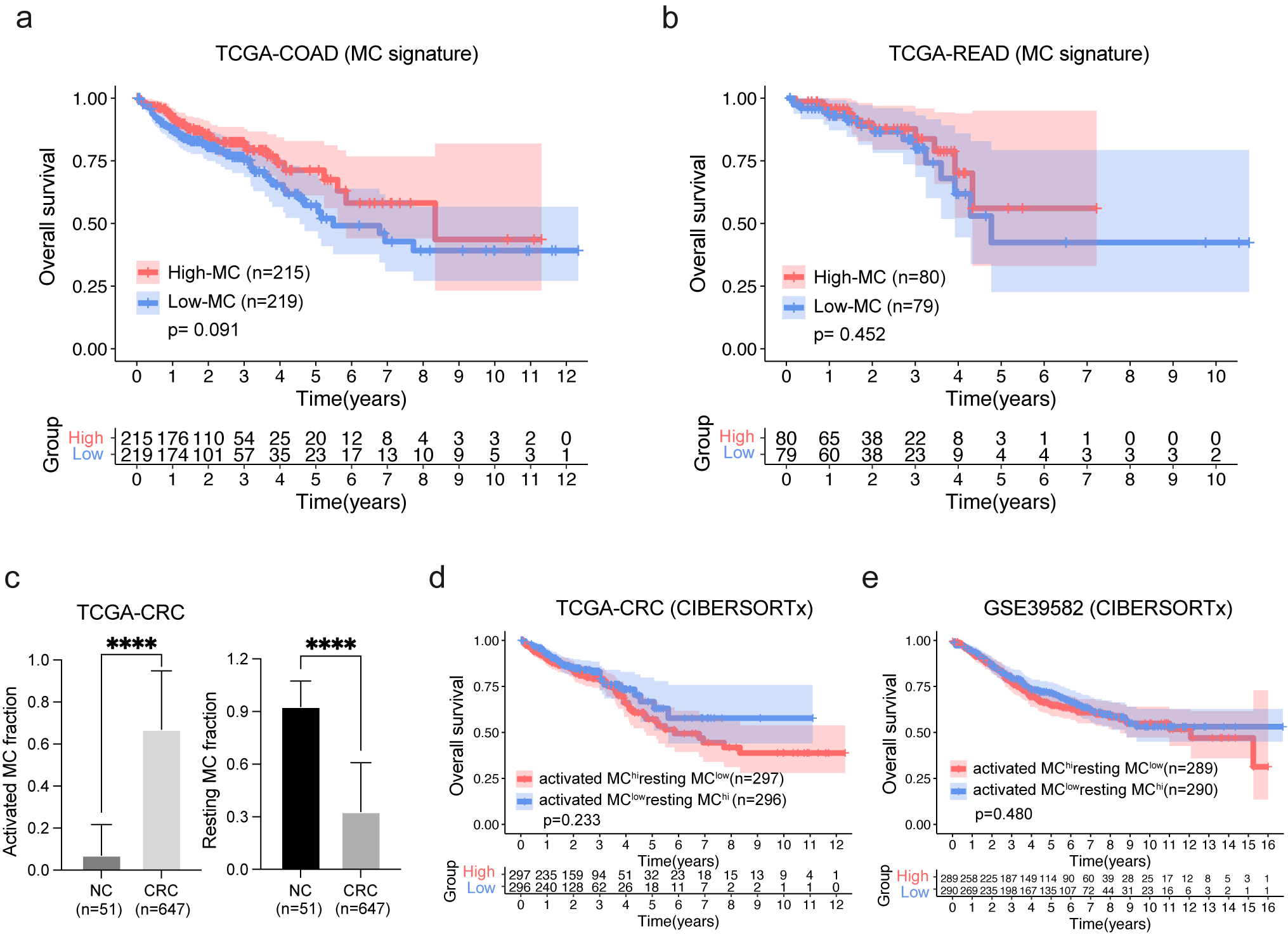

Supplement: Supplementary file 3 — Supplementary Material 3: Figure S3. Supplementary Prognosis Information. (a). Kaplan-Meier overall survival curve of MC signature in TCGA-COAD. (b). Kaplan-Meier overall survival curve of MC signature in TCGA-READ. (c). Comparison of activated MC fraction (left) and resting MC fraction (right) between tumor and normal tissue in TCGA-CRC. Mann-Whitney U test, ****: p < 0.0001. (d). Kaplan-Meier overall survival curve of activated MC fraction in TCGA-CRC. (e). Kaplan-Meier overall survival curve of activated MC fraction in GSE39582. All Kaplan-Meier curves above were generated using median values for grouping. The activated MC fraction and resting MC fraction in c-e were based on results obtained using CIBERSORTx [file 13578_2023_1144_MOESM3_ESM.tif]

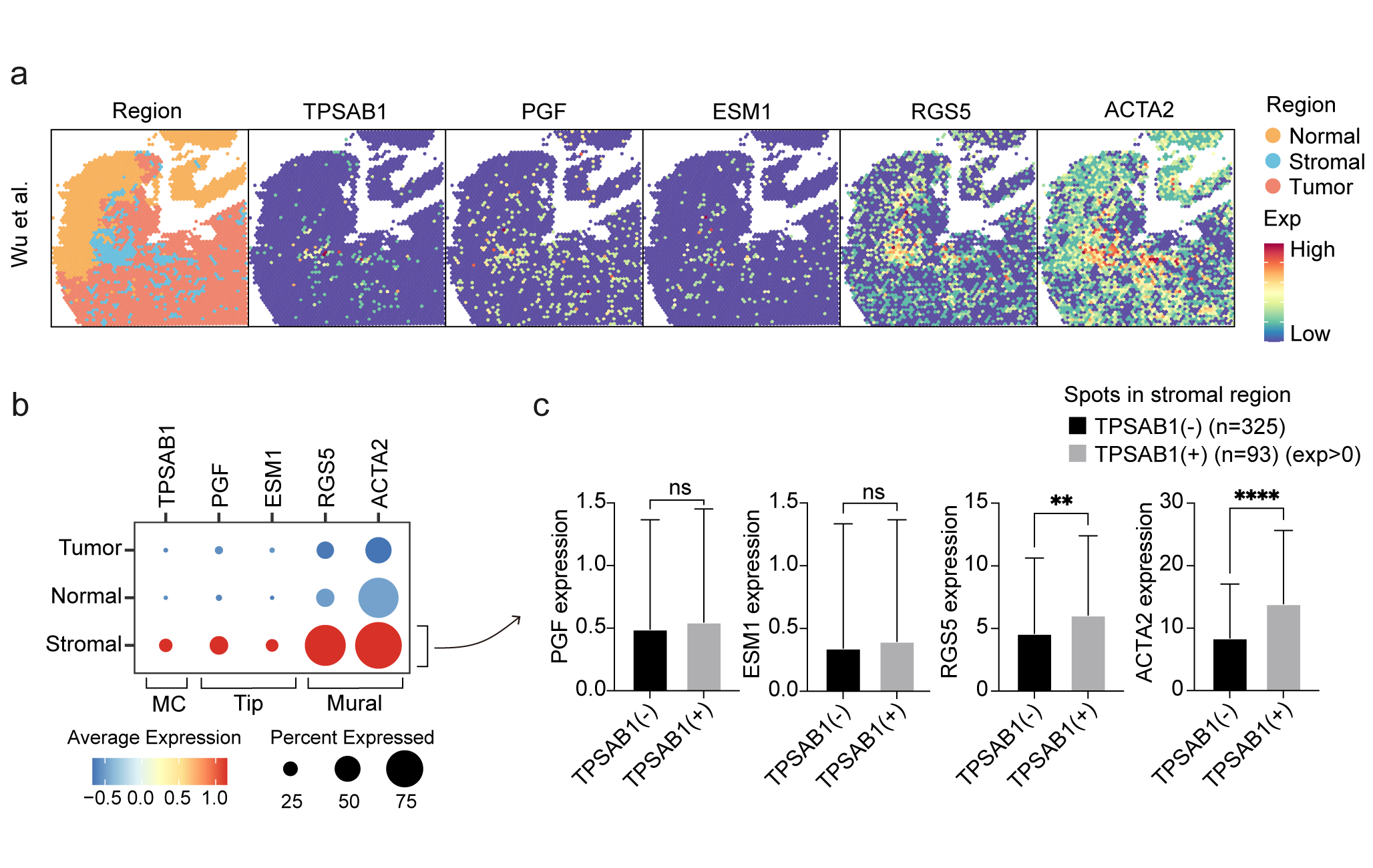

Supplement: Supplementary file 4 — Supplementary Material 4: Figure S4. Spatial co-localization of MCs with mural cells in stromal region. (a). Spatial plots of tissue regions, MCs (TPSAB1), tip cells (PGF and ESM1), and mural cells (RGS5 and ACTA2) in a CRC sample. (b). Dot plots display the expression of MC marker gene, tip cell marker genes, and mural cell marker genes in different tissue regions. (c). Comparison of PGF, ESM1, RGS5, and ACTA2 expressions between TPSAB1- and TPSAB1+ spatial plots in stromal region. Mann-Whitney U test, **: p < 0.01, ****: p < 0.0001 [file 13578_2023_1144_MOESM4_ESM.tif]
